# Assessment of judgment ability in a Brazilian sample of patients with mild cognitive impairment and dementia

**DOI:** 10.1590/1980-57642021dn15-020007

**Published:** 2021

**Authors:** Patrícia Helena Figueirêdo do Vale-Britto, Laura Rabin, Livia Spindola, Ricardo Nitrini, Sonia Maria Dozzi Brucki

**Affiliations:** 1 Behavioral and Cognitive Neurology Unit, Department of Neurology, Cognitive Disorders Reference Center, Hospital das Clínicas, Universidade de São Paulo ‒ São Paulo, SP, Brazil.; 2 Department of Psychology, Brooklyn College and the Graduate Center of the City University of New York ‒ Brooklyn, New York, USA.

**Keywords:** judgment, mild cognitive impairment, Alzheimer disease, frontotemporal dementia, neuropsychological tests, julgamento, disfunção cognitiva, doença de Alzheimer, demência frontotemporal, testes neuropsicológicos

## Abstract

Judgment is the ability to make sound decisions after consideration of relevant information, possible solutions, likely outcomes, and contextual factors. Loss of judgment is common in patients with mild cognitive impairment (MCI) and dementia. The Test of Practical Judgment (TOP-J) evaluates practical judgment in adults and the elderly, with 15- and 9-item versions that require individuals to listen to scenarios about everyday problems and report their solutions.

**Objective:**

Adaptation of TOP-J for a Brazilian sample, preparation of a reduced version and verification of the accuracy of both.

**Methods:**

Eighty-five older adults, including 26 with MCI, 20 with Alzheimer’s disease (AD), 15 with frontotemporal dementia behavioral variant (FTDbv) and 24 controls, underwent neuropsychological assessment including the Brazilian adaptation of the TOP-J (TOP-J-Br).

**Results:**

On both TOP-J-Br versions, controls outperformed MCI, AD and FTDbv patients (p<0.001) and MCI outperformed AD and FTDbv (p<0.001). For the TOP-J/15-Br, the best cutoff for distinguishing controls and patients had a sensitivity of 91.7%, specificity of 59.0% and area under the curve of 0.8. For the TOP-J/9-Br, the best cutoff for distinguishing controls and patients had a sensitivity of 79.9%, specificity of 72.1% and area under the curve of 0.82.

**Conclusion:**

The TOP-J/15-Br, and particularly the TOP-J/9-Br, showed robust psychometric properties and the potential for clinical utility in Brazilian older adults at various stages of neurodegenerative cognitive decline.

## RESUMO.

Julgamento é a capacidade de tomar decisões acertadas, após considerar informações relevantes disponíveis, soluções possíveis, resultados prováveis ​​e fatores contextuais. A perda de julgamento é comum em pacientes com comprometimento cognitivo leve (CCL) e demência. O Teste de Julgamento Prático (TOP-J) avalia o julgamento prático em adultos e idosos, em versões de 15 e 9 itens, que exigem que os indivíduos ouçam cenários sobre problemas cotidianos e relatem suas soluções.

**Objetivo:**

Adaptação do TOP-J para amostra brasileira, elaboração de uma versão reduzida e verificação da acurácia de ambas.

**Métodos:**

Oitenta e cinco idosos, incluindo 26 com CCL, 20 com doença de Alzheimer (DA), 15 com variante comportamental de demência frontotemporal (DFTvc) e 24 controles, foram submetidos à avaliação neuropsicológica, incluindo a adaptação brasileira do TOP-J (TOP-J-Br).

**Resultados:**

Nas duas versões do TOP-J-Br, os controles superaram os CCL, DA e DFTvc (p<0,001) e o grupo CCL superou os grupos DA e DFTvc (p<0,001). Para o grupo TOP-J/15-Br, o melhor ponto de corte para diferenciação entre controles e pacientes apresentou sensibilidade de 91,7, especificidade de 59,0 e área sob a curva de 0,8. Para o TOP-J/9-Br, o melhor ponto de corte para diferenciação entre controles e pacientes teve sensibilidade de 79,9, especificidade de 72,1 e área sob a curva de 0,82.

**Conclusão:**

O TOP-J/15-Br, e particularmente o TOP-J/9-Br, mostraram propriedades psicométricas robustas e o potencial de utilidade clínica em idosos brasileiros em vários estágios de declínio cognitivo neurodegenerativo.

## INTRODUCTION

### Judgment

Judgment can be defined as the ability to make sound decisions after careful consideration of available information, contextual factors, possible solutions and likely outcomes.^1^ ^,^ ^2^ Conceptually, practical judgment is closely related to both problem-solving and decision-making, and while these terms are often used interchangeably in the literature, there are subtle differences. Decision-making refers to the entire process of choosing an action,^3^ while problem-solving involves making probabilistic forecasts about various options to identify the most feasible one(s),^4^ Judgment refers to the components of the decision-making process required to evaluate, estimate, and infer which events will appear and the consequences of each possible outcome as well as the level of satisfaction with potential viable options.^3^ Thus, practical judgment can be considered one of the last stages of the active resolution of a problem. Stating that a person had bad judgment means that he or she made a poor decision after consideration of information, options, and available contextual factors.

From a neuropsychological perspective, judgment relies upon many cognitive processes including memory (remembering relevant past experiences), language (understanding verbal and nonverbal aspects, and communicating the decision to the people involved), sustained attention, reasoning.^5^ ^,^ ^6^ and especially executive functions.^7^ ^,^ ^8^ Emotional and social processes can also impact judgment including one’s level of empathy, sensitivity to social feedback, perception of the consequences of the actions chosen for others, sense of responsibility, and social obligations. It is important, however, not to confuse practical judgment with moral judgment, defined as evaluative judgment of the adequacy of behavior in the context of social perceptions of right and wrong.^9^

Many neuropsychological measures can be used to assess problem-solving including the Wisconsin Card Sort Test (WCST) and the Comprehension subtest of the Wechsler Adult Intelligence Scale (WAIS). Decision-making is often measured by gambling tasks such as the IOWA Gambling Test. However, these measures may not adequately assess the quality of behavior during everyday practical judgment, which requires that an individual actively seek information and determine the relevance of a possible response before reaching a decision.^10^

Among the measures assessing constructs overlapping with practical judgment are the Predicaments Task,^10^ Everyday Problem-Solving Inventory, Reflective Judgment Dilemmas, Practical Problems Test, Everyday Cognition Battery,^5^ Everyday Problems Test, and Everyday Problems Test for Cognitively Challenged Elderly. These tests, however, have been developed for research purposes, have little information on psychometric properties, and are not routinely used by neuropsychologists.^2^ In addition, only the Everyday Problems Test and Everyday Problems Test for Cognitively Challenged Elderly have been studied in older adults with cognitive impairment.^11^

### Judgment assessment

To our knowledge, there are only six judgment tests with psychometric data available for clinical use in older adults: Judgment Questionnaire subtest of the Neurobehavioral Cognitive Status Exam (NCSE JQ), Subscale Troubleshooting Scales of Independent Living, Judgment subtest of the Neuropsychological Assessment Battery (NAB JDG),^12^ Test of Practical Judgment (TOP-J),^1^ Kitchen and Picture Test,^13^ and the Verbal test of practical judgment (VPJ).^14^

Some of these measures mentioned above are limited, however, especially when adapting them for use with clinical samples of adults. Drane and Osato,^15^ for example, observed that the NCSE JQ did not discriminate dementia patients from healthy older adults. Woods and colleagues^7^ found that the NCSE JQ had notable content and statistical problems, including being insensitive to judgment difficulties in Alzheimer’s disease (AD) patients. Furthermore, items on the NAB JDG primarily relate to safety and hygiene issues instead of tapping into high-level judgment dilemmas that require participants to engage in real-world decision-making.^12^

In a recent study, Durant and colleagues^16^ compared estimates of judgment obtained from TOP-J/9 and NAB-JDC in a neurodegenerative disease population. There was a significant amount of inconsistency between these measures, suggesting that they may be measuring different aspects of judgment and would contraindicate using the measures interchangeably. The authors suggests that NAB-JDG may also be more appropriate when there are questions about a patient’s ability to engage in basic hygiene and self-care behaviors and propose that for patients with more advanced disease or greater cognitive impairment, the NAB-JDG may be the more appropriate test, though for patients with more preserved cognition or milder impairments, the TOP-J may be a better choice. When feasible, they recommend that it may be beneficial for these measures to be used in conjunction to obtain more comprehensive information regarding different components of judgment.

The VPJ is a 10-item open-ended scale that was recently developed to ecologically evaluate older adults’ functional domains in which practical judgment is expected to play an important role in successful task performance. These domains were food preparation, shopping, managing medications, handling finances, housekeeping chores, doing laundry, using transportation, and telephone use. VPJ items were constructed to simulate everyday scenarios in which older adults with executive dysfunction may demonstrate poor judgment. The VPJ has demonstrated adequate reliability, strong construct validity, and an optimal VPJ cutoff score for identifying impaired judgment. Although VPJ significantly predicted IADL performance, the IADL skills were not assessed by **“**objective**”** methods, such as direct observation or performance-based measures, and it is not known if it is able to distinguish controls from specific cognitively impaired older patients.^14^

Rabin and colleagues^2^ evaluated neuropsychologists’ practices and perspectives regarding judgment assessment. Participants were 290 doctoral-level members of the International Neuropsychological Society and the National Academy of Neuropsychology who resided in the U.S. or Canada. The tests most frequently reported to assess judgment were WAIS Comprehension, WCST, WAIS Similarities, Delis-Kaplan Executive Function System, and NCSE JQ. The authors discussed that, the three most used measures (Comprehension, WCST, and Similarities) were in fact not developed to assess judgment per se but to evaluate abilities such as novel problem-solving, the understanding of social conventions, and verbal abstraction. Additionally, while the vast majority of respondents indicated that they assess judgment at least ‘‘often’’ during clinical evaluations (and with dementia patients in particular), approximately 90% perceive a need for improved measures for assessing judgment.

#### Test of Practical Judgment

In response to the need for a relevant clinical measure of everyday judgment in older adults, Rabin and colleagues^1^ developed the TOP-J, a questionnaire consisting of 15 (TOP-J/15) or 9 (TOP-J/9) open-ended questions in which participants listen to brief scenarios about daily problems and report aloud their possible solutions. These scenarios were designed to be easily understood and representative of the types of judgment problems encountered by older adults, yet complex enough to require higher-level cognitive abilities. TOP-J items fall broadly within four content domains: safety, social/ethical, financial, and medical.^1^

During administration of the TOP-J, responses are recorded verbatim by examiners. To score the items, examiners check examinee’s response against sample responses listed on the response form; though not an exhaustive list, sample responses encompass a broad range of possible replies. Unusual responses are judged according to their degree of similarity with sample responses in terms of specific content or general meaning. Individual responses are scored on a 4-point scale (0, 1, 2, or 3 points), with higher numbers indicating better judgment. Total scores are obtained by summing the items (range 0-45 for the TOP-J/15 and 0=27 for the TOP-J/9).

Table 1 provides a summary of published studies employing the TOP-J. Notably, the TOP-J appears to be sensitive to diagnostic group differences in individuals with preclinical and mild dementia^1^ and shows associations with gray matter density in prefrontal regions (left inferior and superior frontal gyri). Pickens and colleagues^17^ conducted a review of articles published from 2003 to 2009, to identify the most effective measure to evaluate executive functions in adults with cognitive impairment, determined via the psychometric properties of the measures. Among the 18 measures included, only the TOP-J included all statistical tests required for the development of a scale, considering factor analysis, validity and reliability.

**Table 1.** Summary of TOP-J data.

Published studies	Sample	Test	Results
Rabin et al.^1^	n=134: 14 AD, 34 amnestic MCI (aMCI), 39 with normal cognition but significant cognitive complaints (CC), and 35 controls (HC)	TOP-J/9	HCs obtained higher scores than CC, MCI, and AD, while ADs obtained lower scores than HC, CC, and MCI participants (approximately 2 *SD*s below the mean of HCs). CC and MCI participants showed an intermediate level of performance (approximately 1 *SD* below the mean score of HCs). Cronbach's alpha=0.63, test-retest stability=0.78, interrater reliability=0.95.
Rabin et al.^2^	n=120 (13 AD, 34 aMCI, 34 CC and 39 HC)	TOP-J/9	Investigated the relationship between gray matter density and judgment controlling for age, education, gender, intracranial volume, verbal memory, and crystallized knowledge. Lower TOP-J scores were associated with reduced gray matter density in prefrontal regions (left inferior and superior frontal gyri).
Selected abstracts			
Rabin et al.^27^	n=210 (43 MCI, 62 CC, 105 HC)	TOP-J/15	Re-evaluated validity of the TOP-J in a sample of older adults with different demographic characteristics than the original normative sample. HCs obtained higher TOP-J scores than MCI (CC and MCI did not differ). A notable finding was the 2- to 3-point (approximately 0.5 SD) reduction in scores among HC and MCI in the current (urban) as compared to the original (rural) sample.
Baldock et al.^39^	n=18 (9 AD and 9 FTD)		Results revealed a statistically significant difference in performance between AD and FTD patients, matched on the basis of overall MoCA scores.

aMCI: amnestic MCI; MoCA: Montreal Cognitive Assessment; SD: standard deviation.

In sum, the TOP-J appears to provide valuable information about everyday judgment, which can be used for diagnostic purposes and to address clinical questions related to functional competence and possible interventions. Unfortunately, to our knowledge there is no judgment test widely utilized with Brazilian older adults in various stages of neurodegenerative cognitive decline. Therefore, the goal of the current study was to evaluate judgment using the TOP-J/Br (Brazilian adaptation) in a sample of older adult controls and in those with preclinical dementia (i.e., mild cognitive impairment; MCI), AD, and the frontotemporal dementia behavioral variant (FTDbv). We additionally sought to determine key psychometric properties of the TOP-J in this diverse sample (e.g., aspects of validity, reliability, and sensitivity and specificity).

## METHODS

### Material

The sample consisted of 85 older adults divided into four groups: controls and patients with MCI, AD, or FTDbv. For all groups, we defined inclusion criteria as age greater than or equal to 50 years and schooling greater than or equal to 4 years. We defined exclusion criteria as visual disturbances and/or hearing without correction, musculoskeletal disorders that could impact testing, history of alcoholism or other substance dependence, neurological disorders aside from dementia and MCI, those using psychoactive drugs (e.g., antipsychotics), not compensated chronic diseases, and scores greater than or equal to 6 on the short for of the Geriatric Depression Scale (GDS-15). We did not exclude patients with depression treated with stable doses of antidepressants for three months. For AD and FTDbv patients, we permitted the use of medications (such as antipsychotics and selective serotonin reuptake inhibitors) to control cognitive and behavioral changes. Antipsychotics were allowed in AD and FTD patients at stable doses and with controlled symptoms, without psychotic symptoms according to the attending neurologist.

We evaluated 61 patients in total. Twenty-six patients had MCI (amnestic or not amnestic, single- or multiple-domain according to Winblad and colleagues’ criteria).^18^ Thirty-five patients had mild dementia, subdivided into two groups: 20 with probable diagnosis according to the criteria of the National Institute of Neurological and Communicative Disorders and Stroke and Alzheimer's Disease and Related Disorders Association (NINCDS-ADRDA)^19^ and mild intensity according to the criteria of the Diagnostic and Statistical Manual of Mental Disorders, 4th edition, revised (DSM-IV),^20^ and 15 diagnosed with DFTbv^21^ and mild intensity according to the DSM-IV.^20^

The control group consisted of 24 community volunteers (without memory complaints and with independent activities of daily living). Based on the above criteria, we excluded potential control participants with the following test scores: Mini Mental State Examination (MMSE) scores less than the median for education,^22^ scores lower than or equal to 5 on delayed recall of the Brief Cognitive Battery (BCB),^23^ scores greater than 3.41 on the Informant Questionnaire of Cognitive Decline in the Elderly (IQCODE),^24^ and scores greater than 2 on the Functional Activities Questionnaire (FAQ).

This study was approved by the Ethics Committee for Research Project Analysis (CAPPesq) of clinical directors of HCFMUSP. All participants received detailed information about the study and signed a consent form.

### Instruments

All participants underwent the TOP-J-Br in addition to cognitive screening tests (MMSE and BCB), functional assessment (FAQ and IQCODE), and evaluation of depression (GDS-15). Participants also completed a comprehensive neuropsychological assessment including: MapZoo, Trail Making Test (TMT), Verbal Fluency Test to phonemic categories, Digit Span subtest of the Wechsler Intelligence Scale for Adults (WAIS-III), Wisconsin Card Sorting Test short version (WCST),^25^ Comprehension subtest of the WAIS-III, Rey Figure test, Rey Auditory Verbal Learning Test (RAVLT), and the Logical Memory subtest of the Wechsler Memory Scale (WMS-R).

Patients were evaluated and diagnosed by members of the Behavioral and Cognitive Neurology Group of the Department of Neurology and Cognitive Disorders Reference Center, Hospital das Clinicas, University of São Paulo School of Medicine. Physicians provided the diagnostic classifications, and the neuropsychological assessment was performed by a neuropsychologist (PHFV) who was not blinded to patient diagnosis, in one session, taking approximately 90 minutes.

### Brazilian adaptation of TOP-J

For the Portuguese language adaptation process, we followed the procedures developed by Guillemin and colleagues.^26^ First, the translation from English into Portuguese was performed by two researchers, independently. The two translations were compared, and some adjustments were made on items without cultural or functional correspondence in Brazil. The adaptations resulted in a consensus version between investigators in which modifications were made to items 1, 2, 4, 8, 11, 14 and 15 of the 15-item version of the TOP-J. For example, in Brazil, blood pressure pills can be bought without prescription, so in Question 1, this term was replaced by controlled pills. In Question 2, social security number was replaced by individual registration number. Next, a pilot study was conducted with this consensus version, which was applied to 10 older adults without cognitive complaints, to evaluate the understanding of the items. Subsequently, another round of modifications was made to items 1, 4, 13 and 15. The items were judged by the authors with experience in cognitive assessment and related diseases. The resulting TOP-J translation was then submitted to back-translation by an individual fluent in English and compared to the original version. This resulted in several additional changes to items 4, 7, 8, 10 and 12 and a final version referred to as the TOP-J/Br.

### Statistical analysis

Descriptive statistics were calculated for all variables. Because the Kolmogorov-Smirnov normality assumption was not met for the TOP-J, analyses to compare means utilized non-parametric tests (i.e., Kruskal-Wallis followed by the Bonferroni method). The chi-square test was used to assess the association between qualitative variables. Pearson correlation coefficients were used to assess the degree of linear relationship between quantitative variables.

A factor analysis was carried out using the principal component analysis with varimax rotation to determine the underlying structure of the TOP-J test and potentially to eliminate items with low loadings. The KMO index (Kaiser's Measure of Sampling Adequacy) and Bartlett's sphericity test were used to determine whether the factor analysis was relevant to the dataset.

Internal consistency analysis consisted of Cronbach’s alpha coefficient. Sensitivity and specificity analyses used receiving operating characteristic (ROC) curves. Statistical analyses were performed using the *Statistical Package for the Social Sciences* (SPSS) v.19.0 program. The significance level was set to 5% (p<0.05) for all tests.

## RESULTS

### Descriptive analysis of sample characterization

Table 2 presents the participants’ sociodemographic characteristics. The groups were statistically equivalent with respect to gender and education but not regarding age. Specifically, the MCI group and AD group were older than individuals in the control group and FTDbv group.

**Table 2.** Sociodemographic profile and TOP-J/Br results

	Gender (n=85)		Age		Education		TOP-J/9	TOP-J/15
	Male	Female	Mean(SD)	Median	Mean(SD)	Median	Mean(SD)	Mean(SD)
Controls	8	16	65.5 (7.5)	65	12.3(3.05)	13	20.2 (2.4)^A,B,C^	33.4 (4.0)^A,B,C^
MCI	10	16	71.6(5.52)	72	10.4(6.51)	9	17.7 (3.5)^A,D,E^	30.15 (4.88)^A,D,E^
AD	9	11	75.1(6.24)	77	9.3 (5.19)	8	14.8 (4)^B,D^	26.5 (4.9)^B,D^
FTDbv	12	3	65.3(8.68)	67	10.3(6.08)	8	13.3 (4.9)^C,E^	24.2 (6.47)^C,E^

*Kruskal-Wallis test followed by Bonferroni test. ^A^Control≠MCI; ^B^Control≠AD; ^C^Control≠FTD; ^D^MCI≠AD; ^E^MCI≠FTD.

### Validity

#### Validity based on internal structure

Prior to performing the factor analysis TOP-J/15-Br, we performed two tests to determine whether components would result from the analysis. Specifically, we used the KMO of sampling adequacy and Bartlett's sphericity test. Results indicated that the sample was adequate for carrying out the exploratory factor analysis (KMO=0.64; >0.5 and Bartlett’s sphericity test: p=0.000, <0.05; test statistic 179.63 and 105 degrees of freedom).

Subsequently, we performed an exploratory factor analysis and identified five factors that could explain 54% of the variability of the data. The factors had eigenvalues greater than one. This value is similar to that found in the study of Rabin et al. in 2013,^27^ where six factors were identified, with eigenvalues ​​greater than 1 and accounting for 55.9% of total variance.

On the basis of the study of the original TOP-J,^1^ only the first factor was considered to identify the factor loadings lower than 0.4. Thus, six items could be excluded from TOP-J/15-Br. The reduced version (TOP-J/9-Br) then comprised the remaining nine items (1, 2, 4, 6, 7, 8, 10, 12 and 14). The items 4, 6, 7, 8, 10, 12 and 14 were then rearranged to 3, 4, 5, 6, 7, 8 and 9.

Table 3 illustrates these modifications to the TOP-J.

**Table 3.** Exploratory factor analysis to a single factor.

Item	Domain	Factor loadings of TOP-J/15-Br	Factor loadings of TOP-J/9-Br	Factor loadings of original TOP-J/9
**TOP-J 1**	**Medical**	**0.43**	**0.36**	**0.62**
**TOP-J 2**	**Financial**	**0.49**	**0.57**	**0.43**
TOP-J 3*	Safety*	0.23*	n/a*	1.10
**TOP-J 4**	**Financial**	**0.51**	**0.55**	**0.60**
TOP-J 5*	Social/ethical*	0.39*	n/a*	n/a
**TOP-J 6**	**Medical**	**0.77**	**0.79**	**0.68**
**TOP-J 7**	**Safety**	**0.43**	**0.46**	**0.45**
**TOP-J 8**	**Social/ethical**	**0.49**	**0.52**	**0.37**
TOP-J 9*	Safety*	0.38*	n/a*	n/a
TOP-J 10	Social/ethical	0.50	0.54	n/a
TOP-J 11*	Safety*	0.34*	n/a*	n/a
**TOP-J 12**	**Social/ethical**	**0.41**	**0.35**	**0.41**
TOP-J 13*	Medical*	0.15*	n/a*	n/a
TOP-J 14	Social/ethical	0.53	0.58	n/a
TOP-J 15*	Financial*	0.33*	n/a*	0.49

*Items not included in TOP-J /9-Br; Items in bold are common to the TOP-J/9 original and TOP-J/9-Br; The items are numbered according to their position on the TOP-J/15 protocol.

With the reduction in the number of TOP-J/Br items, the administration time also decreased. In general, the test with 15 items takes approximately 15 minutes to administer while the reduced version takes approximately 10 minutes.

#### Evidence for criterion-related validity

We next examined group differences in TOP-J-Br scores. Table 2 shows that there were statistically significant difference in the total score of the TOP-J/15-Br and TOP-J/9-Br between control and MCI, control and AD, control and FTDbv, MCI and AD, and MCI and FTDbv. There was no statistically significant difference between AD and FTDbv.

#### Analysis of the evidence of validity for related construct (convergent and discriminant)

From here on, all reported analyses are for the 9-item TOP-J version, except where otherwise noted.

In the TOP-J/9-Br, it was observed that in the control group there was a statistically significant positive correlation between the total score and the Comprehension test (R=0.51; p<0.01). Evidence of divergent validity emerged by the lack of correlation with the measure of depression.

### Reliability

#### Internal consistency

The TOP-J/9-Br has achieved good internal consistency (Cronbach's alpha=0.68).

### Sensitivity and specificity

Table 4 shows that the TOP-J/9-Br had good accuracy to discriminate controls from patients with MCI, AD and FTD, controls from patients with dementia (AD and FTDbv) and controls from patients (MCI, AD and FTDbv).

**Table 4.** Cutoffs for TOP-J/Br.

Group	Area under curve	Cutoff	Sensitivity %	Specificity %
Control versus MCI	0.73**	19	79.2	57.7
Control versus AD	0.87*	18	83.3	70.0
Control versus FTD	0.90*	18	83.3	73.3
Control versus dementia	0.89*	18	83.3	71.4
Control versus patient	0.82*	19	79.2	72.1

*p<0.001; **p<0.05.

## DISCUSSION

The TOP-J was developed for use with older adults and assesses judgment ability related to safety, medical, financial, and social/ethical issues. Items were initially developed on the basis of a careful review of the literature on judgment and related constructs, information from older adult participants in the Dartmouth Memory and Aging Study and their spouses, and a perceived need in the field of neuropsychology.^1^ ^,^ ^2^ For the current study, we translated the TOP-J into Portuguese and adapted it for use with Brazilian older adults (with a lower overall level of education than in the initial standardization sample by Rabin and colleagues). Overall, the Brazilian version of the TOP-J demonstrated adequate psychometric properties and clinical utility.

### Validity based on internal structure

#### Reduction of the scale

TOP-J/15-Br scores were adequate for carrying out the exploratory factor analysis. Reduction of items to generate the Brazilian scale was carried out in a manner consistent with the original work,^1^ which opted for the extraction of a single factor by identifying items with factor loadings less than 0.4, which can be presumed irrelevant to the overall construct. As in the original study, we also identified six items with factor loadings below 0.4, which were then removed. Items retained for the TOP-J/9-Br covered all the domains proposed by the authors, though they were not identical to the items in the original TOP-J/9.^1^ This is not surprising given the cultural and other differences between the current and previous participant groups.

#### Criterion validity

Criterion validity for the TOP-J/9-Br was adequate. For the total TOP-J/9-Br score, there was a statistically significant difference in performance between controls and MCI, controls and AD, controls and FTDbv, MCI and AD, and MCI and FTDbv. There were no statistically significant differences between AD and FTDbv groups.

Notably, MCI patients performed significantly worse than controls on the TOP-J-Br. Studies have shown that patients with MCI often present with executive dysfunction and reduced functional activities of daily living that involve complex reasoning.^28^ ^,^ ^29^ There is increasing evidence of problem-solving difficulties in MCI as measured by IADL scales or traditional neuropsychological tests,^29^ ^,^ ^30^ ^,^ ^31^ decision-making measured by gambling tests, and on the Everyday Problems Test.^32^ Our results are consistent with the initial validation study of the TOP-J/9,^1^ in which the controls had significantly lower performance than MCI and AD, and MCI had significantly lower AD performance than AD. In a study conducted with the TOP-J/15 original within a demographically diverse sample of older adults,^27^ controls also demonstrated better performance than MCI.

Rabin and colleagues^1^ found that the NCSE was not able to distinguish MCI individuals from controls. This reinforces the idea that the detection of subtle declines in judgment depends on the sensitivity of the measure used.^30^ Perhaps an assessment approach that combines a measure of practical judgment with tests of executive functioning, including aspects of complex reasoning would offer a strong screening tool.^13^ Indeed, in their 2008 survey, Rabin and colleagues^2^ reported that neuropsychologists generally agreed that judgment should be assessed via a combination of approaches, particularly clinical interviews with the patient, neuropsychological tests, and informant interviews.

Although patients with dementia may perform some routine tasks relatively properly, problem-solving skills related to work and social and home environments are often affected. In unstructured situations, executive dysfunction could lead to poor judgment in everyday situations, such as impulsive decisions, inadequate exploitation of relevant issues, cognitive rigidity, or judgment based on consideration of immediate consequences only.^33^

It was interesting, but not surprising, that there was no statistically significant difference between AD and FTD patients. The TOP-J is designed to be ecologically representative of problems routinely encountered by older adults, involving few emotional processes as compared to higher-order cognitive processes. In this context, it is important not to confuse practical judgment, assessed by the TOP-J, with moral or social judgment, which is related to the ventromedial cortex and amygdala,^34^ ^,^ ^35^ and typically more affected in patients with FTD than with AD.

In FTDbv, there is great prominence of frontal circuit dysfunction (e.g., medial orbital) in the early stages, with posterior involvement of the dorsolateral circuits. Classical cognitive tests commonly used to evaluate dementia are sensitive to dorsolateral functions (and this seems to be the case with the TOP-J). Such measures may not enable differential diagnosis of early FTDbv,^36^ whereas tests evaluating medial orbital functions of social cognition, including recognition of basic emotions, social decision-making, inferences about the mental states of others, and awareness of social behavior and moral judgment are better suited to detect the early disease stages.^9^ ^,^ ^36^

As an example, in a study by Mendez and colleagues,^35^ moral judgment was initially evaluated with the Moral Behavior Inventory,^37^ in which individuals are asked to mark 1 - not wrong, 2 - slightly wrong, 3 - moderately wrong, and 4 - very wrong, to items such as fails to keep promises, driving after drinking, takes the biggest piece of a pie, and asks others to do part of their housework. Subsequently, patients were evaluated with two trial dilemmas proposed by Greene and colleagues,^34^ for example ''Imagine a trolley runaway is approaching five workers who will die if it continues. You are on a footbridge over the tracks, between the trolley and five workers. Next to you on the catwalk there is a stranger. The only way to save the lives of five workers is to push this stranger off the bridge for his body to stop the trolley. One person will die if you do this, but the five workers will be saved. Do you push the stranger to save five workers?" AD patients outperformed those with FTD on this task, which usually taps into moral behaviors or the ability to be ethical and accept norms and rules.^35^ The fact that the performance of individuals with AD was similar individuals with FTD could suggest that, as desired, the TOP-J taps more cognitive than emotional processes, unlike the moral judgment tests.

Many studies have been devoted to investigating decision-making skills in FTD^36^ ^,^ ^38^ as compared to cognitive impairment of different etiologies. Only one study, however, verified the impairment of practical judgment in patients with FTD. In a recently published abstract, Baldock and colleagues^39^ found a statistically significant difference in the TOP-J scores of the groups of 9 AD patients and 9 FDT patients, matched according to overall MOCA scores. FTD patients had a mean score of 15.89 and AD patients had a mean score of 20.89 (cutoff for “normal” scores is 19.4). However, the authors did not mention whether the groups were matched for education or dementia severity. It is possible that the observed group differences were due to the fact that participants had moderate or severe dementia, when the clinical differences become more obvious.

#### Construct validity

Convergent validity for the TOP-J/9-Br was established through significant correlations with WAIS Comprehension scores and a lack of correlation with scores on a self-report measure of depression. This supports evidence of discriminant validity, also observed by Rabin and colleagues,^1^ and confirms that the TOP-J taps more cognitive than emotional aspects of practical judgment. Perhaps, if the sample had been larger, we would have observed correlations between the TOP-J and other executive functioning, language, or memory tests.^1^

#### Reliability

Internal consistency for the TOP-J/9-Br, estimated by Cronbach's alpha, was 0.68. This value is within the acceptable range for cutoff scores of tests in social sciences, ranging from 0.6 to 0.8 and is consistent with the value of 0.63 obtained for the TOP-J/9 in the 2007 study of Rabin and colleagues. Moreover, items seem to be measuring a single construct, as the alpha values ​​for each of the items were not significantly greater than the overall value.

Cronbach's alpha values for the TOP-J/9-Br and for the original study ​​compare favorably with Cronbach's alpha of 0.45 for NAB JDG,^12^ with Cronbach's alpha values for NCSE JQ of 0.04 and 0.46 found respectively for controls and patients with AD,^7^ and with a value of 0.07 found in the NCSE JQ by Rabin and colleagues.^1^ MacDougall and Mansbach^40^ found a Cronbach's alpha value of 0.83 for the NAB JDG. Despite being a high value, data were collected from residents in nursing homes and assisted living facilities, which may limit the generalizability of their findings. Furthermore, the authors did not include control subjects. Mansbach and colleagues^13^ found alpha values of 0.88 and 0.93 for the Kitchen and Picture Test in studies with samples of 121 and 163 older adult participants, respectively. Again, these data may not be generalizable because they utilized individuals in long-term care facilities. Moreover, according to the authors, the test scores have an element of subjectivity (due to the nature of the test) and health care professionals with varying levels of training and experience participated in the administration and scoring

#### Sensitivity and specificity

The overall accuracy of a test can be described as the area under the curve.^41^ In the current study, we chose to prioritize a higher sensitivity value, with the highest specificity possible, to decrease the chance of false negative error. A sensitive measure (i.e., a test that is generally positive in the presence of the disease) must be chosen as the consequences of a deficit not identified are considerable, as in case of dementia patients with judgment deficits, who could persist in behaviors that are not safe. Patients and their families can then be educated about the nature and consequences of impaired judgment and the relationship of observed symptoms to the disease process.^1^ Sensitive tests are also useful in the early stages of a diagnostic process,^41^ as in MCI patients.

In the case of TOP-J/Br, the cutoff points with high sensitivity suggest that individuals with judgment problems will have a high probability of being identified. However, because the specificity was lower, some patients with impaired judgment may not have been identified. Therefore, we recommend a detailed history and a comprehensive neuropsychological assessment for cases in which, although the test has shown impaired judgment, the clinical opinion is that practical daily judgment is preserved.

It is worth mentioning that the TOP-J is intended to evaluate practical judgment and not to be used as a general dementia screening tool or diagnostic test in its own right. To our knowledge, this is the first study to report on the sensitivity and specificity of both versions of the TOP-J. A study of the NAB JDG, demonstrated a sensitivity of 0.61 and specificity of 0.88, to distinguish AD patients from patients with unspecified diagnoses, without a control group.^42^ In another study with the Kitchen Picture Test, the cutoff represented a sensitivity of 0.85 and specificity of 0.72, but the authors did not specify the types of dementia or other clinical subgroups.^13^

From a clinical perspective, we recommend a cutoff score of 19 for the TOP-J/9, to differentiate individuals with impaired judgment from those with preserved judgment.

For the identification of an early disturbance of judgment in the Brazilian population, with a brief assessment, the TOP-J/9 should be used as a screening measure as it showed good sensitivity and specificity already in the comparison between control and MCI, with the same sensitivity and higher specificity in the comparison between controls and patients with varying degrees of cognitive impairment.

To our knowledge, the TOP-J is the first clinical judgment test to be evaluated in a population of elderly Brazilian individuals with an average low level of education. Additionally, the current study is the first to investigate the accuracy (sensitivity and specificity) performance of FTDbv patients in a judgment ability test and to compare their functioning to AD and MCI patients. The Brazilian version of TOP-J showed adequate psychometric properties and was able to distinguish the clinical groups. We recommend its use in clinical settings in Brazil with a possible role in capacity assessments for legal purposes.
